# Insights on epithelial cells at the single-cell level in hepatocellular carcinoma prognosis and response to chemotherapy

**DOI:** 10.3389/fphar.2023.1292831

**Published:** 2023-11-17

**Authors:** Wenqian Qi, Qian Zhang

**Affiliations:** Department of digestive, China-Japan Union Hospital of Jilin University, Changchun, China

**Keywords:** hepatocellular carcinoma, epithelial cells, differentiation, scissor algorithm, risk assessment system, prognosis, drug therapy

## Abstract

**Background:** Hepatocellular carcinoma (HCC) originates from Epithelial cells, and epithelial lineage plasticity has become a promising research direction for advancing HCC treatment. This study aims to focus on Epithelial cells to provide target insights for detecting HCC prognosis and response to drug therapy.

**Methods:** Single-cell RNA sequencing (scRNA-seq) data from GSE149614 were clustered using Seurat, and the differentiation and evolution of epithelial cells were analyzed by Monocle 2. Scissor+ and Scissor− Epithelial cells associated with the prognostic phenotypes of bulk RNA-seq of HCC were screened using the Scissor algorithm for differential analysis to screen candidate genes. Candidate genes were overlapped with prognostic related genes screened by univariate Cox regression, and the Least Absolute Shrinkage and Selection Operator (LASSO) sparse penalty was imposed on the intersection genes to construct a risk assessment system.

**Results:** Eight major cell subpopulations of HCC were identified, among which the proportion of epithelial cells in non-tumor liver tissues and HCC tissues was significantly different, and its proportion increased with advanced clinical stage. During the progression of HCC, the whole direction of epithelial cells differentiation trajectory was towards enhanced cell proliferation. Differential analysis between Scissor+ and Scissor− epithelial cells screened 1,265 upregulated and 191 downregulated prognostic candidate genes. Wherein, the upregulated genes were enriched in Cell processes, Genetic information processing, Metabolism and Human disease with Infection. Nevertheless, immune system related pathways took the main proportions in downregulated genes enriched pathways. There were 17 common genes between upregulated candidate genes and prognostic risk genes, of which CDC20, G6PD and PLOD2 were selected as components for constructing the risk assessment system. Risk score showed a significant correlation with tumor stage, epithelial-mesenchymal transition (EMT) related pathways and 22 therapeutic drugs, and was an independent prognostic factor for HCC.

**Conclusion:** This study revealed the cellular composition of HCC, the differentiation evolution and functional landscape of epithelial cells in the further deterioration of HCC, and established a 3-gene risk model, which was closely related to clinical features, EMT, and drug sensitivity prediction. These findings provided insights in patient prognosis and drug therapy detection for HCC.

## Introduction

Hepatocellular carcinoma (HCC) is a major liver tumor that threatens the lives of many people with poor prognosis around the world, accounting for 5% of all incident cases and 8.4% of all deaths due to cancer worldwide (Moon et al., 2020; Shu et al., 2022; Alqurashi et al., 2023; Romualdo et al., 2023). The little improvement in prognosis of HCC patients over the past 15 years is mainly attributed to inadequate monitoring of risk individuals and advanced tumor manifestations, with no effective treatment to achieve long-term survival of patients (Ogunwobi et al., 2019; Ahn et al., 2021; Huang et al., 2023). New biomarkers are needed to improve HCC detection, prognosis, treatment response prediction, and disease monitoring during therapy.

A healthy liver is static on mitosis, but after toxic injury or resection, cells can quickly enter the cell cycle to restore liver quality and function (Campana et al., 2021). Indeed, there is a high degree of plasticity among Epithelial cells, hepatocytes and biliary epithelial cells in mammalian liver, which is related to the mechanism of tissue repair mechanisms and liver lesions such as cancer (Johnson, 2019). During HCC progression, the transition of epithelial cells to a mesenchymal phenotype exacerbates the motility and invasiveness of various epithelial cell types (Jayachandran et al., 2016). In this context, the scientific community believes that advances in the field of Epithelial cell plasticity may hold great promise for the development of therapies for HCC patients (Ko et al., 2020). It is also worth mentioning that the concept of numerous markers of Epithelial cells as therapeutic targets for liver cancer has been demonstrated in preclinical studies, such as CXCL5 combing with CXCR2 promoted epithelial-mesenchymal transition (EMT) via PI3K/Akt/GSK-3β/Snail signaling (Xia et al., 2015; Zhou et al., 2015), Keratin 1 (Ogunnigbagbe et al., 2022), p63, the isoforms of which were related to tumor recurrence and reduced survival (Gonzalez et al., 2017), E-cadherin (Nakag et al., 2014), EpCAM, a latent marker for cancer stem cells, was reported to be intensely correlated with unfavorable clinical prognosis in HCC (Gerlach et al., 2018; Park et al., 2020). However, the cellular and molecular mechanisms of Epithelial involvement in HCC remain unclear.

Single cell RNA sequencing (scRNA-seq) technology based on high-throughput sequencing at a single-cell resolution has been widely recognized as feasible and advantageous in dissecting tumor heterogeneity and analyzing the cellular mechanism and differentiation of important cell subsets (Ho et al., 2019). For example, a recent study in bladder cancer correlated scRNA-seq data disclosed the relationship between molecular and clinical features, guiding molecular diagnosis and targeted therapy (Robertson et al., 2017). In the meantime, scRNA-seq is also applied to assess the effectiveness and safety of new drugs in clinical trials (Srivatsan et al., 2020). In this study, we aimed to explore the heterogeneity of HCC at the single-cell level, select epithelial cells to explore their differentiation evolution, and combine the analysis of bulk RNA-seq data of HCC to identify novel biomarkers of HCC to predict prognosis and response to drug therapy.

## Materials and methods

### Study omics of human liver cancer tissues

The omics data downloaded in this study included scRNA-seq omics data and bulk RNA-seq omics data. ScRNA-seq omics data were obtained from the Gene Expression Omnibus (GEO) database under accession number GSE149614, which gives 70,000 single-cell transcriptomes for 10 HCC patients from four relevant sites: primary tumor, portal vein tumor thrombus (PVTT), metastatic lymph node and non-tumor liver. The bulk RNA-seq omics data were obtained from three different data sources, one was the TCGA-LIHC dataset (n = 342) in The Cancer Genome Atlas (TCGA) database (https://portal.gdc.cancer.gov/) (Yan et al., 2022), one was the ICGC-LIRI-JP dataset (n = 212) from the Hepatocellular Carcinoma Database (HCCDB) database (http://lifeome.net/database/hccdb/), another one was the GSE14520 dataset (n = 221) from the GEO database.

### Standard processing and clustering of scRNA-seq

ScRNA-seq data were read with the use of the Seurat package (Butler et al., 2018), and only cells with a gene number between 200 and 8,000 and a mitochondrial gene proportion of less than 10% were retained. SCTransform function was utilized to normalize the single-cell UMI count data based on a negative binomial regression model with regular parameters to remove the influence of deep sequencing. The RunHarmony function was used to set default parameters to integrate the transformed data and correct for batch effects. Then, with the aid of RunUMAP function, the dimension of Uniform Manifold Approximation and Projection (UMAP) was reduced, and FindClusters was used to perform graph-based clustering on the neighbor graph constructed by the FindNeighbors function call. The parameters of the two functions were set to dims = 1:30 and resolution = 0.05, respectively. The cell identity of each cluster was obtained by referring to the cell marker resources provided by the CellMarker database.

### Construction of pseudotime trajectory

Monocle 2 applies recently developed machine learning strategy that was described as reversed graph embedding (RGE) to accurately reconstruct complex single-cell trajectories (Qiu et al., 2017). We used monocle2 to read the count data of epithelial cells in the expression matrix, merged the phenotype information of the cells, used newCellDataSet function to construct cds objects, and filtered out the genes expressed in less than 10 cells. Then, the differentialGeneTest function (fullModelFormulaStr = "∼ site") was adopted to calculate the differentially expressed genes (DEGs) between the primary tumor and metastatic samples of HCC patients. Using the reduceDimension function and setting the parameters max_components = 2, method = "DDRTree” to reduce the dimension, and under the control of the orderCells function, the cells were sorted and the trajectory was constructed. In particular, the branch of the primary tumor was set here as the starting point of the trajectory.

### Differential expression analysis between trajectories branches

Monocle 2 provides Branched Expression Analysis Modeling (BEAM) method to analyze the branching of cell data and designated nodes to identify DEGs related to branching, and heatmap was generated using plot_genes_branched_heatmap to identify DEGs associated with clades. The plot_genes_branched_pseudotime function was also applied to visualize the expression of the interested genes at the branch point.

### Capture of prognostic associated cells

Scissor is a method for quantifying the similarity between single-cell and batch data by measures such as Pearson’s correlation for each pair of cells and batch samples to identify cell subsets from single-cell data associated with a given phenotype (Kearnes et al., 2014; Sun et al., 2022). The novelty of Scissor lies in its use of phenotypic information from bulk data to identify highly correlated cell subpopulations with diseases, thereby revealing disease mechanisms and improving disease diagnosis and treatment. The bulk expression matrix of TCGA-LIHC, ICGC and GSE14520 and the single-cell expression matrix of GSE149614 were input in Scissor to screen out the cell subsets related to prognostic phenotypes in HCC patients, and further classified them into Scissor+ and Scissor-cell subgroups.

### Differential expression analysis of Scissor groups based on subgroups associated with prognostic phenotypes

The FindMarkers function in Seurat was used to identify DEGs between Scissor+ and Scissor− cell subgroups. The R package RobustRankAggreg (Ogunwobi et al., 2019) then integrated the rankings of logFC values to obtain a comprehensive ranking list for each bulk RNA-seq cohort. Genes that showed the same trend of differential expression in the three cohorts and had an RRA score less than 0.05 were considered as candidate genes.

### Construction of regression model

Univariate Cox regression of clinical survival data was performed in the three bulk RNA-seq cohorts to screen genes with *p* < 0.05 as prognostic related genes for HCC. The common prognostic risk genes (HR > 1) in the three bulk RNA-seq cohorts were used for overlap analysis with the candidate genes. LASSO sparse penalty was imposed on the overlapping genes, with high confidence to select the most important genes for the prognosis of HCC to build a risk regression model.

### Drug sensitivity analysis of the risk regression model

The expression matrix of HCC in the TCGA-LIHC cohort was used as training data to predict drug response using the R package oncoPredict. The correlation between the half maximal inhibitory concentration (IC50) values of the resulting drugs and the risk regression model was analyzed by Spearman correlation test, in which the false discovery rate (FDR) was adjusted by Benjamini and Hochberg, and FDR <0.05 and | cor | > 0.5 was defined as a significant correlation.

### Statistical analysis

All statistical analyses were processed using R software. Functional enrichment analyses were processed in clusterProfiler. The analysis to evaluate the regression model included Kaplan-Meier survival analysis and Receiver Operating Characteristic (ROC) analysis, which were performed by the “survival” package and “pROC,” respectively. Differences between two groups of continuous variables were assessed using the Wilcoxon rank-sum test, and differences between more than two groups of variables were compared using the Kruskal–Wallis test. The *p*-value for significance in this study was set at < 0.05.

## Results

### Single-cell landscape of human HCC

**FIGURE 1:**
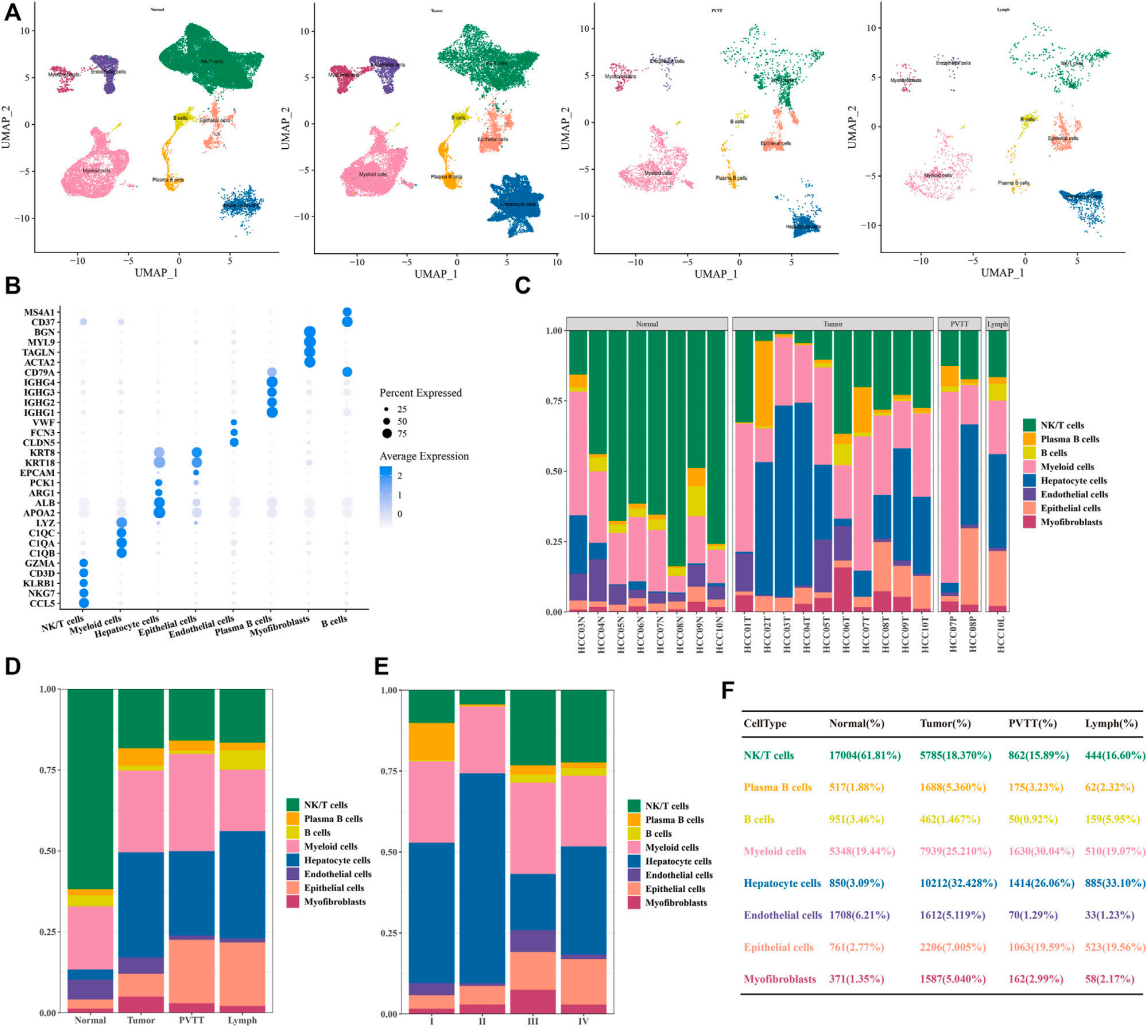
Single-cell profiles of human HCC **(A)** Visualization of UMAP plot of 8 types of cells in non-tumor liver, primary tumor, PVTT, and metastatic lymph node tissue. **(B)** Bubble plot of marker gene expression of cells with different identities. **(C)** The proportion of cells with eight identities in each tissue. **(D)** Proportional distribution of cells with eight identities in different HCC tissue types. **(E)** The percentage of cells with 8 identities was judged according to clinical stage grouping. **(F)** Statistical table of the proportion of cells with 8 identities in different HCC tissue types.

After the standard processing pipeline of scRNA-seq, cell clustering and annotation, a total of eight types of cell clusters were identified in the HCC of GSE149614. In terms of the distribution of 8 types of cells in different tissues of different origins, non-tumor liver distributed 27,510 cells, primary tumor distributed 31,491 cells, portal vein tumor thrombus (PVTT) distributed 5,426 cells, and portal vein tumor thrombus (PVTT) distributed 5,426 cells, the metastatic lymph nodes distributed 2,674 cells (Figure 1A). Each has its own specifically expressed genes, shown in Figure 1B. After our statistical analysis, we found that the proportion of NK/T cells in primary tumor, PVTT and metastatic lymph node tissues was greatly reduced compared with non-tumor liver tissues. However, the proportion of hepatocyte cells and epithelial cells increased significantly (Figures 1C, D, F). Differences in the distribution of cell types were also observed among stages I-IV, and the proportion of epithelial cells increased with the advance of clinical stage (Figure 1E). Through the single-cell landscape analysis we revealed epithelial cells seemed to occupy an important position in the development of HCC.

### Differentiations of epithelial cell subsets in the progression of hepatocellular carcinoma

**FIGURE 2:**
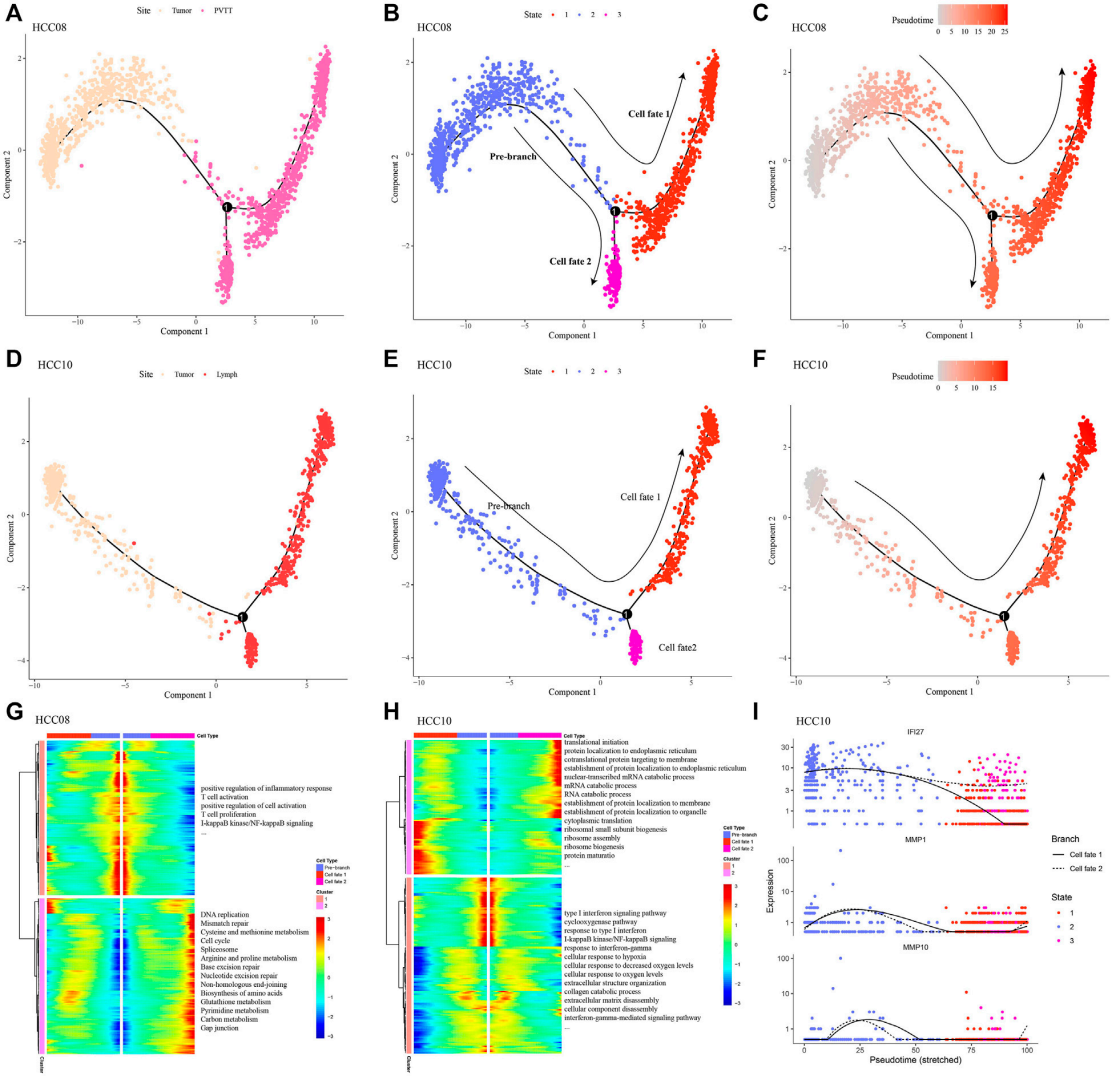
Differentiation of epithelial cell subsets in the progression of hepatocellular carcinoma **(A–C)** Epithelial differentiation trajectories during the evolution from primary tumor to PVTT, colored according to site, cell state, and pseudotime, respectively. **(D–F)** Epithelial differentiation trajectories during progression from primary tumor to metastatic lymph nod, colored according to site, cell state, and pseudotime, respectively. **(G)** The expression trend and enriched biological processes of DEGs between the branches of epithelial cells produced in the evolution from primary tumor to PVTT. **(H)** Changes in the expression and enriched biological processes of DEGs between the branches of epithelial cells produced during the development from primary tumor to metastatic lymph nod. **(I)** Expression profile of DEGs between branches along epithelial differentiation trajectories.

Given the difference in the proportion of epithelial cells between non-tumor liver tissues and HCC tissues, and the tendency for the proportion to increase with clinical stage, ee extracted epithelial cells from the two HCC samples with the highest percentage of epithelial cells, primary tumor of HCC08, primary tumor and metastatic lymph nodes of PVTT and HCC10 were included. Monocle2 was used to explore the dynamic changes of gene expression in epithelial cells during the development and progression of HCC, it was found that there were two different fates of epithelial cells in the evolution from primary tumor to PVTT (Figures 2A–C): One of them showed a gradual weakening of inflammatory response, T cell activation and proliferation, and the other showed a gradual strengthening of cell proliferation and metabolism (Figure 2G). Epithelial cells in the process of developing from primary tumor to metastatic lymph nod also had two differentiation trajectories (Figures 2D–F): one was the gradual improvement of RNA catabolic, protein localization and protein translation ability, the other one was a tendency to weaken the strength of the immune response and the cellular response to hypoxia (Figure 2H). We also found that the expression of IFI27, a differential gene between branches, was significantly higher in the differentiation pathway along cell fate 2 than in the differentiation pathway along cell fate 1. Two others branched DEGs, MMP1 and MMP10, were also presented during the differentiation of epithelial cells from primary tumor to metastatic lymph nod (Figure 2I). These findings confirmed the action of Epithelial cells on cell proliferation, and forced us to propose a hypothesis that Epithelial cells may have an inhibitory effect on inflammatory response targeting HCC.

### Identification of prognostic associated epithelial cell populations and genes

**FIGURE 3:**
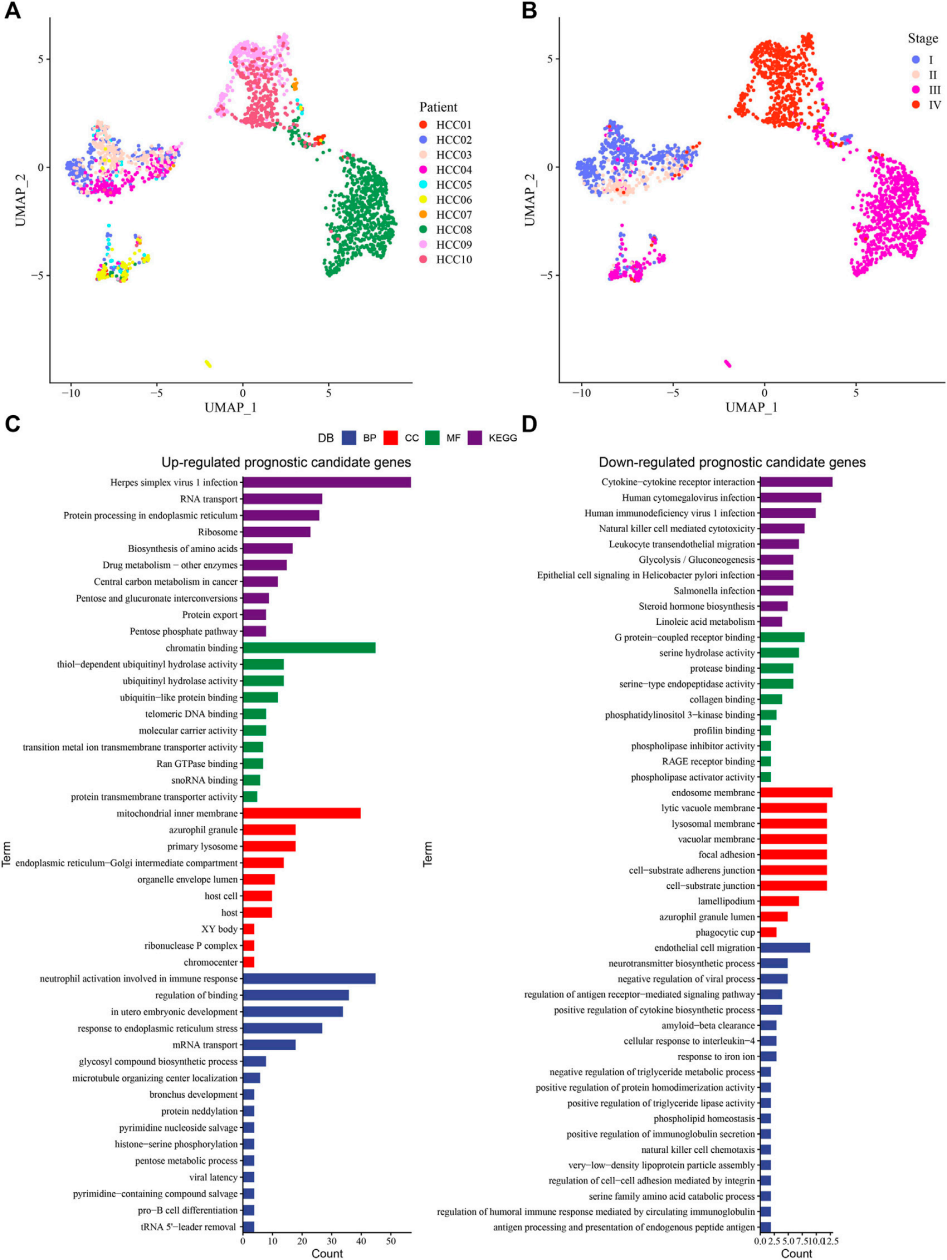
Identification of prognostic associated epithelial cell populations and genes **(A)** The UMAP plot of the epithelial cells stained according to the origin of sample. **(B)** UMAP plot of epithelial cells colored according to sample stage. **(C)** Functional enrichment analysis of 1,265 upregulated prognostic candidate genes. **(D)** Functional enrichment analysis of 191 downregulated prognostic candidate genes.

The epithelial cells in each primary tumor were selected and labeled according to their stage, and we found that the samples in stage Ⅰ mainly included HCC01, HCC02 and HCC03. HCC04 was at stage Ⅱ, HCC05, HCC06, HCC07 and HCC08 were at stage Ⅲ, and HCC09 and HCC10 were at stage Ⅳ (Figures 3A, B). Using the Scissor algorithm, we identified prognostic phenotypes-related Scissor+ and Scissor-epithelial cells, including 447 Scissor + cells and 184 Scissor-cells associated with the prognostic phenotypes of TCGA-LIHC, 673 Scissor + cells and 131 Scissor-cells associated with prognostic phenotypes of ICGC, 877 Scissor + cells and 10 Scissor-cells associated with prognostic phenotypes of GSE14520. Differential analysis between Scissor+ and Scissor− epithelial cells screened 1,456 DEGs, including 1,265 upregulated prognostic candidates and 191 downregulated prognostic candidates. We found that the upregulated genes were mainly enriched in Cell processes (cell cycle), Genetic information processing (RNA transport, Ribosome, etc.), Metabolism (Biosynthesis of amino acids) and Human disease with Infection (Herpes simplex virus 1 infection). As for enriched pathways with downregulated genes, immune system related pathways (Cytokine-cytokine receptor interaction, Leukocyte transendothelial migration) and Human disease with Infection (Human cytomegalovirus infection and Human immunodeficiency virus 1 infection, etc.) took the main proportions. Collectively, the most significantly correlated pathways were those favoring cancer progression (Figures 3C, D).

### Derivation and validation of a prognostic assessment formula

**FIGURE 4:**
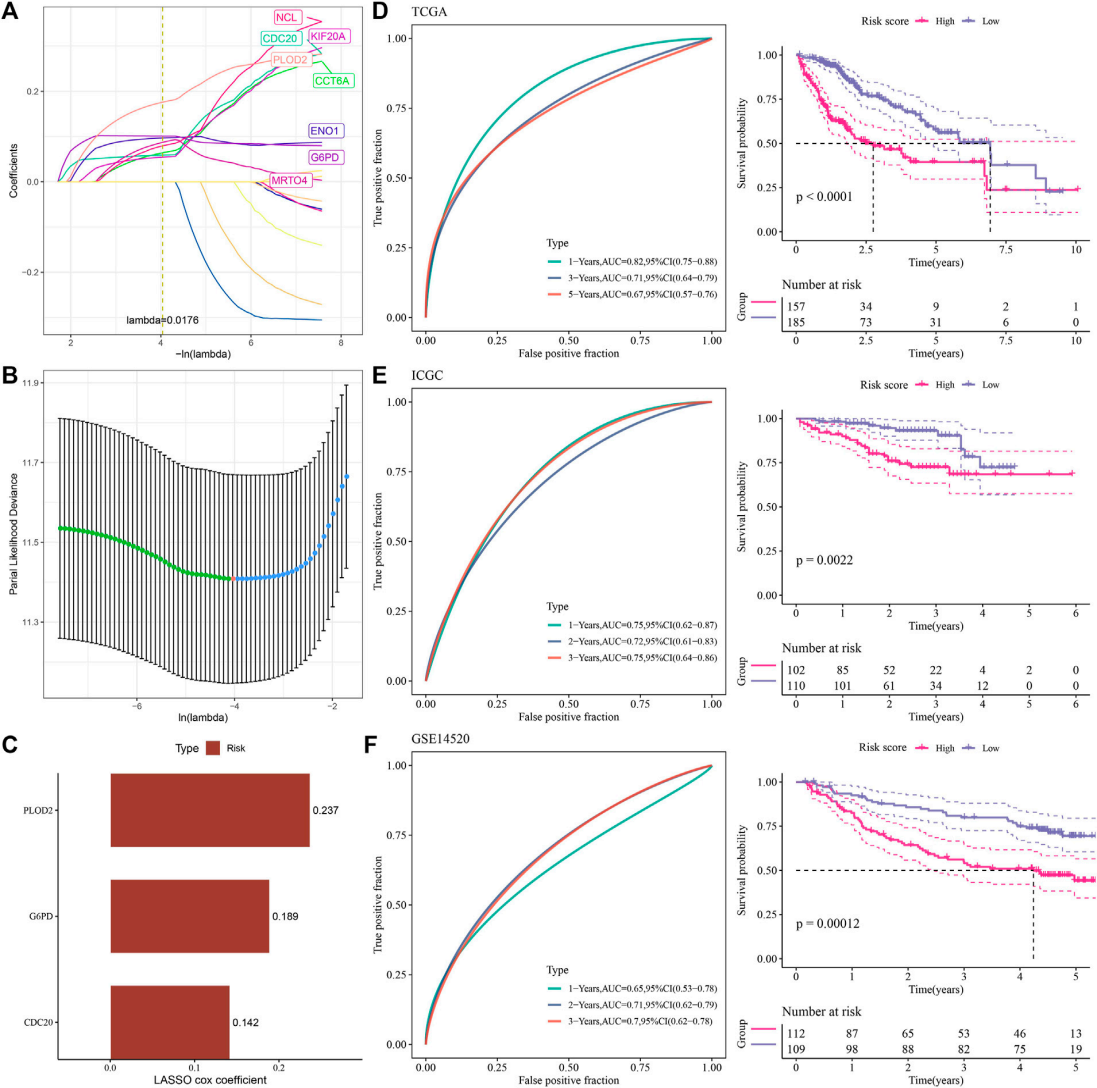
Derivation and validation of a prognostic assessment formula **(A,B)** LASSO sparse penalty was imposed on 17 gene. **(C)** The LASSO Cox coefficients of 3 genes selected by stepwise multifactor regression analysis. **(D)** ROC analysis and Kaplan-Meier survival analysis of the prognosis evaluation system in the TCGA-LIHC cohort. **(E)** ROC analysis and Kaplan-Meier survival analysis of the prognostic evaluation system in the ICGC cohort. **(F)** ROC curve and Kaplan-Meier curve of the prognostic evaluation system for the prognosis of patients in the GSE14520 cohort.

Using univariate Cox regression analysis, we obtained prognostic risk genes in the TCGA-LIHC, ICGC and GSE14520 cohorts, which were overlapped with 1,265 upregulated prognostic candidate genes, and 17 genes were found in the overlapping part, including NCAPG, NCL, DBF4, ENO1, KIF20A, RAN, MRTO4, CDC20, CDK1, UBE2C, CCNB1, STMN1, CCT6A, DLGAP5, G6PD, SSB, PLOD2. LASSO sparse penalty was imposed on them, and eight genes had nonzero coefficients (Figures 4A, B). In order to obtain the most parsimonious model with adequate fitting degree, stepwise multivariate regression analysis was performed, and three genes (CDC20, G6PD and PLOD2) were selected as components for the construction of risk assessment formula (Figure 4C). LASSO gave the coefficient for each gene, and the risk formula was: Risk Score = +0.1424668*CDC20 + 0.1886310*G6PD+0.2367155 *PLOD2. The risk score was calculated in the bulk RNA-seq cohort of each HCC and prognosis was predicted. The risk score showed a significant inverse correlation with overall survival (OS). The accuracy of risk score in predicting prognosis was also tested by ROC curve. For 3-year OS, the area under the ROC curve (AUC) of the TCGA-LIHC, ICGC, and GSE14520 cohorts were 0.71, 0.75, and 0.7, respectively, indicating high prognostic accuracy of the model (Figures 4D–F).

### Risk score and clinical characteristics in predicting the prognosis of HCC and the relationship between them

**FIGURE 5:**
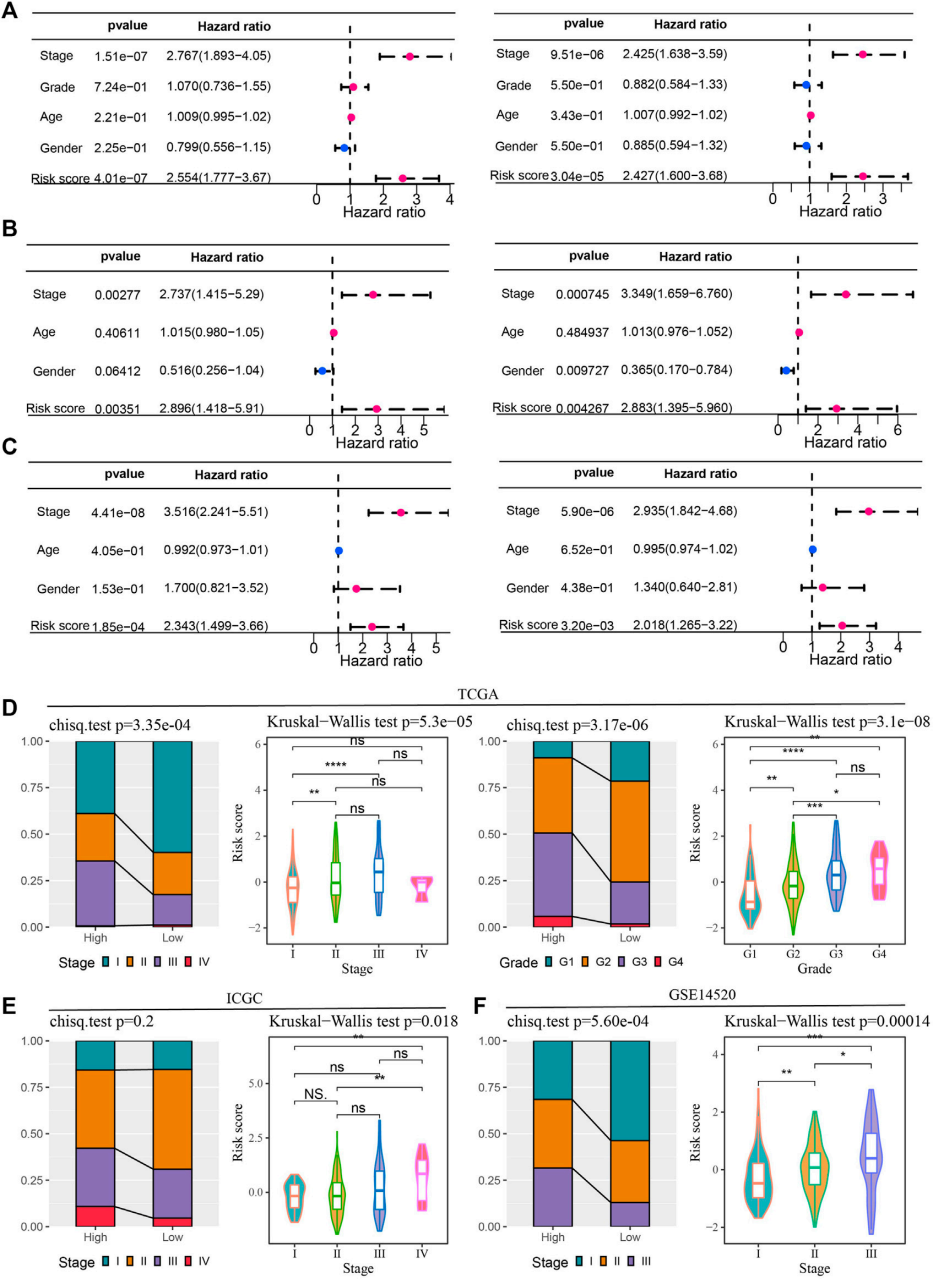
Risk score and clinical characteristics in predicting the prognosis of HCC and the relationship between them **(A–C)** Univariate and multivariate Cox regression analyses of clinical characteristics and risk scores in the TCGA-LIHC dataset, ICGA and GSE14520 cohorts. **(D)** Bar and violin plots to explore the association of risk score with stage and grade in TCGA-LIHC. **(E)** Stage distribution of high-risk and low-risk groups in the ICGC dataset and risk scores of samples in each stage. **(F)** Stage of samples grouped by risk score and risk score of samples grouped by stage in the GSE14520 dataset.

Univariate and multivariate Cox regression analyses of risk score and clinical factors were performed in 3 bulk RNA-seq cohorts of HCC to determine independent variables predicting HCC prognosis. Among the four clinical variables and risk scores provided by the TCGA-LIHC dataset, stage and risk score were independent variables that could independently predict HCC prognosis (Figure 5A). Univariate and multivariate Cox regression analysis based on clinical data and risk score in ICGA identified stage, gender and risk score as independent prognostic factors for HCC (Figure 5B). The conclusion obtained in the GSE14520 cohort was the same as that in the TCGA-LIHC dataset, that stage and risk score were independent prognostic predictors of HCC (Figure 5C). By correlating these independent prognostic variables, risk score and stage in TCGA-LIHC were found influence each other, and the proportion of stage Ⅲ samples in the high-risk group was significantly higher than that in the low-risk group. The risk score of samples in stage Ⅲ was significantly higher than that in stage Ⅰ. Grade also showed a significant correlation with risk score. The proportion of G3-G3 patients composing the high-risk group was significantly higher than that of G3-G4 patients composing the low-risk group. Risk score were analyzed in each grade, and it was found that the risk scores of samples in G3 and G4 groups were significantly higher than those in G1 and G2 groups (Figure 5D). The high-risk and low-risk groups in the ICGC dataset did not show significant differences in stage distribution. However, the risk score of stage Ⅳ samples was significantly higher than that of stage Ⅰ-stage Ⅱ samples (Figure 5E). In the verification set GSE14520, stage was very significantly associated with risk score. Specifically, the proportion of stage Ⅲ in the high-risk group was significantly higher than that in the low-risk group, and the risk score increased with the increase of risk score (Figure 5F).

### Association of risk score with key biological processes occurring in cancer progression

**FIGURE 6:**
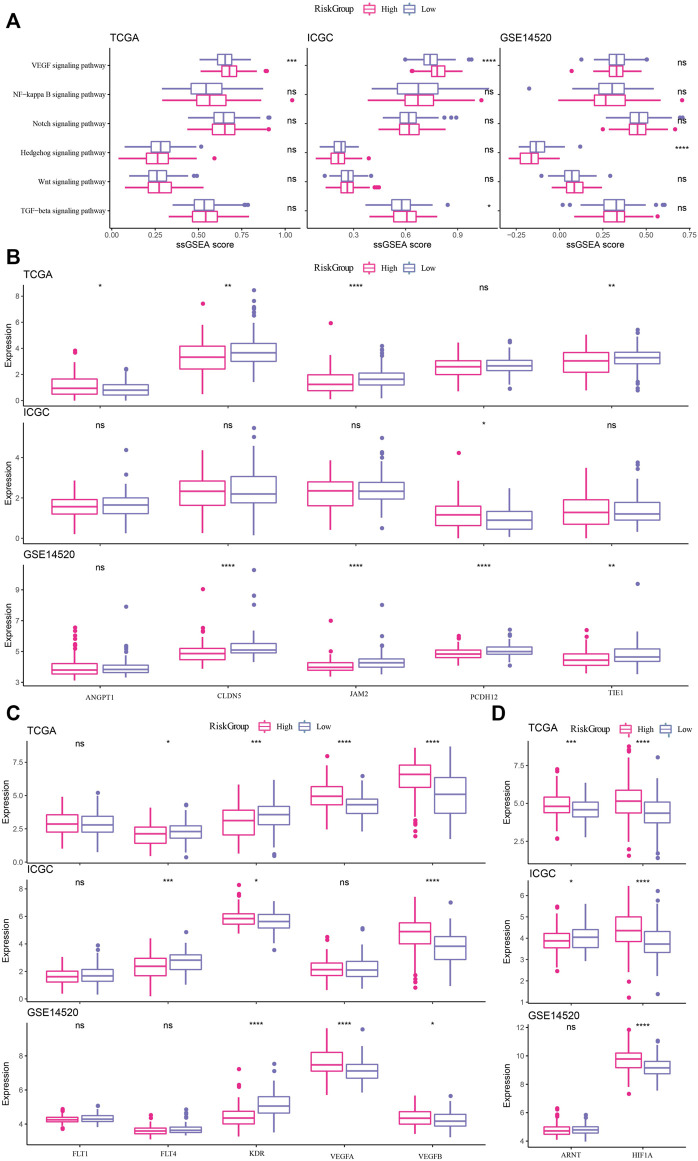
Association of risk score with key biological processes occurring in cancer progression **(A)** Relationship between EMT-related pathways and risk score in TCGA-LIHC, ICGC and GSE14520 datasets. **(B)** Relationship between vascular stability-related genes and risk score in TCGA-LIHC, ICGC and GSE14520 datasets. **(C)** Differential expression of VEGF-related genes between high-risk and low-risk samples in the three datasets. **(D)** Differential expression analysis of Hypoxia-related genes between high-risk and low-risk samples in the three datasets. * and ns denote *p*-value, * <0.05, * *p < 0.001, *p < 0.0001, the ns was no significant difference.

The association between the risk score and pathophysiological events or carcinogenic factors occurring during cancer progression, including epithelial-mesenchymal transition (EMT), vascular stability, and hypoxia, was investigated. We found that EMT related VEGF signaling pathway displayed significant enrichment score between high and low risk groups (Figure 6A). Then, the expression levels of VEGF-related genes were compared between high-risk and low-risk samples in the three datasets. VEGFA and VEGFB were more expressed in high-risk samples, and FLT4 was more expressed in low-risk samples (Figure 6C). CLDN5, JAM2 and TIE1 in vascular stability-related genes also showed significant negative correlation with risk score in TCGA-LIHC and GSE14520 datasets. Paradoxically, PCDH12 showed opposite trends in ICGC datasets and GSE14520 datasets, positively correlated with risk score in the former dataset, and negatively correlated with risk score in the latter dataset (Figure 6B). As a close connection between VEGF signaling pathway and Hypoxia in cancer development (Florentin et al., 2022), Hypoxia-related genes were also compared between high-risk and low-risk samples, and it is certain that HIF1A expression was significantly higher in high-risk samples than in low-risk samples (Figure 6D). Based on the above results, it was speculated that the risk score may partly affect the cancer progression.

### The relationship between risk score and drug therapy

**FIGURE 7:**
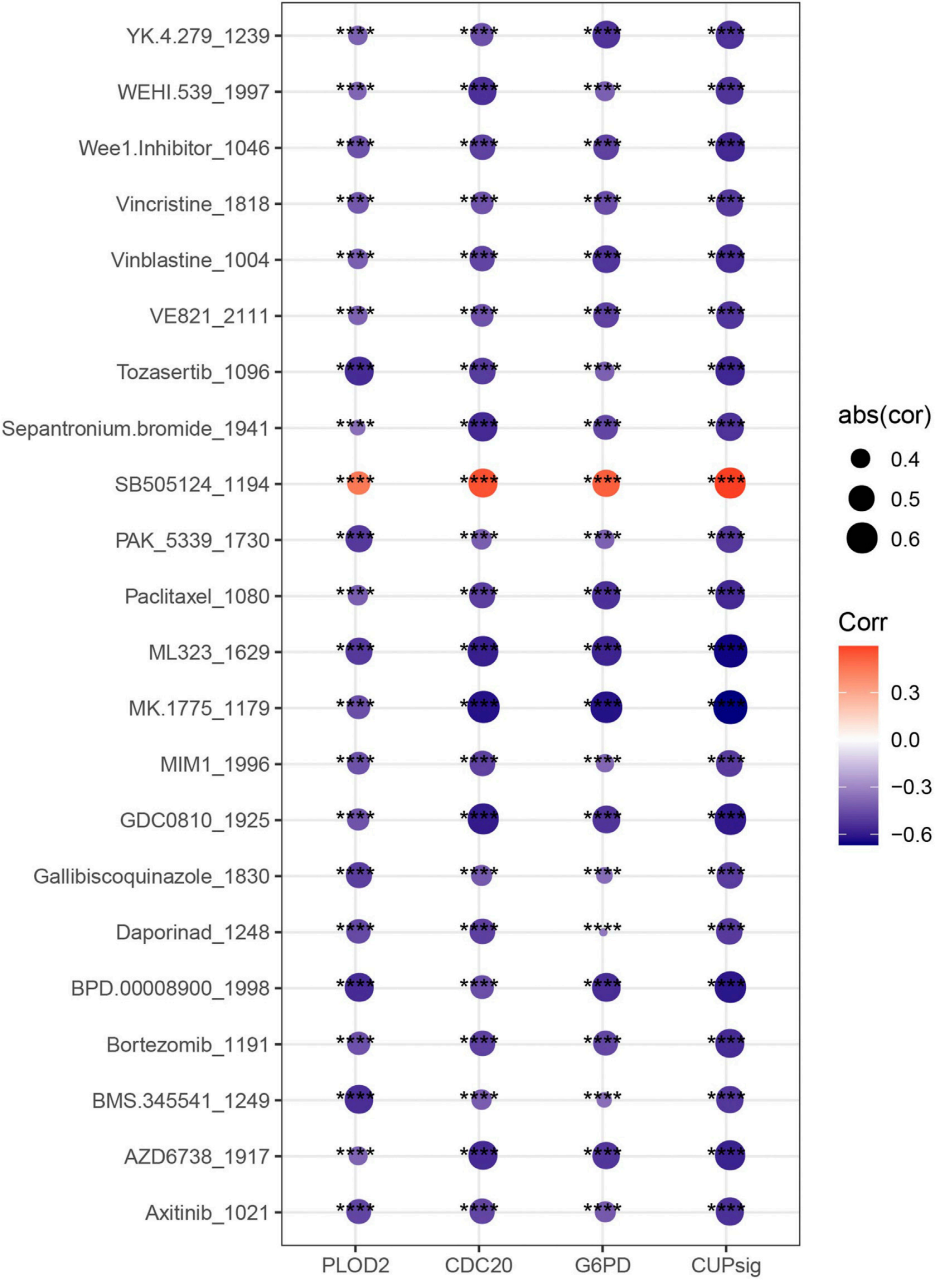
Correlation between risk score and IC50 value of drugs.

The IC50 value of each drug in the samples in TCGA-LIHC was calculated, and a total of 22 drugs were found to be significantly correlated with risk score. The IC50 values of 21 drugs were significantly negatively correlated with risk score, which may be more suitable for the treatment of HCC patients with high-risk score such as Vincristine, Vinblastine, Paclitaxel, Daporinad, Bortezomib and Axitinib, which are commonly used chemotherapy drugs for HCC. The IC50 value ofSB505124, an inhibitor for TGFβ receptor was more suitable for the treatment of patients with low-risk score (Figure 7).

## Discussion

HCC originates from Epithelial cell population and consists of tumor cells, basement membrane, and surrounding stroma (Holczbauer et al., 2022; Yao et al., 2023). The phenotype appears to be closely associated with specific gene mutations, tumor subsets, and/or oncogenic pathways (Calderaro et al., 2019). In this study, we first focused on the composition landscape of cells in HCC under its tissue background, and emphasized the differentiation trajectory and functional influence of Epithelial cells, which was driven by the great regenerative potential of the liver, and its repair ability was mainly attributed to the ability of differentiated Epithelial cells, hepatocytes and biliary Epithelial cells to proliferate after injury (Ko et al., 2020).

The discovery of the hepatocyte profile will help decipher the molecular and cellular mechanisms that drive the healthy liver into disease states, provide insights into the detection of novel therapeutic targets, and pave the way for effective disease interventions (Ma et al., 2021). We deciphered the heterogeneity of HCC at the single cell level and identified 8 cell types. The proportion of Epithelial cells in HCC tissues increased with the increase of clinical stage. We also detected two distinct fates of epithelial cells during the evolution from primary tumor to PVTT, one showing a trend toward progressively diminished immune activity and the other showing a trend toward increased proliferative and metabolic capacity of the cells similar to Heparanase/Syndecan-1 Axis (Yu et al., 2022). Epithelial cells during the progression from primary tumor to metastatic lymph nod also had two differentiation trajectories, one phenomenon was the gradual improvement of RNA catabolic, protein localization, and protein translation, the other was the attenuation of immune response and cellular response to hypoxia, which reflects the dynamic pathophysiological regulation of Epithelial differentiation in the deterioration of HCC.

Techniques such as single-cell omics in combination with molecular and functional studies will help reveal the remaining unknowns in this field (Gadd et al., 2020). This study linked scRNA-seq analysis with the molecular and functional studies of bulk RNA-seq to explore more effects of epithelial cells on HCC using an algorithm called Scissor, which has not been widely used in combined scRNA-seq and bulk RNA-seq studies. We found the prognostic candidate genes in each bulk RNA-seq dataset, and finally screened 3 upregulated prognostic candidate genes to develop the risk assessment system. Cell division cycle 20 homologue (CDC20) is a cell cycle regulator that controls the correct segregation of chromosomes during mitosis (Bruno et al., 2022). CDC2 has oncogenic properties and also regulates anticancer drug responses, and is considered an emerging target for cancer therapeutic intervention (Jeong et al., 2022; Wavelet-Vermuse et al., 2022) Additionally, CDC20 was reported to be related to immune infiltration in cancer. For example, a positive relationship of CDC20 with the infiltration of CD8^+^ T cells, CD4^+^ T cells, as well as natural killer cells in HCC was observed (Lai et al., 2021; Xiong et al., 2021). These findings indicated that CDC20 could be a promising target for HCC therapy in terms of cell cycle inhibition or studying tumor immune vaccine (Wang et al., 2022). Glucose-6-phosphate dehydrogenase (G6PD) is a rate-limiting enzyme in pentose phosphate pathway (PPP), which induces EMT by activating signal transducer and activator of transcription 3 (STAT3) pathway, thus promoting the metastasis of HCC cells (Lu et al., 2018). G6PD also has the properties of vascular regulation and participates in the metabolic adaptation of hypoxia (Baptista et al., 2022). More importantly, G6PD related inhibitors are being studied for cancer treatment. We believed that this newly found gene in HCC may also expand the treating method for HCC. PLOD2 expression was identified as a significant, independent factor of poor prognosis by the study of Noda et al. (2012). PLOD2 is induced by hypoxia and affects chemotherapy resistance in biliary tract cancer patients through EMT (Okumura et al., 2018). Moreover, several pharmacological inhibitors of PLOD2 have been proved to have anti-metastatic effects (Du et al., 2017). Collectively, these three genes are prospective drug research targets for HCC treatment. In the future, the expression levels of these three genes on mRNA and protein should be validated first combined with exploring their functions on cell cycle, metastasis or metabolism under hypoxia environment. In the present study, the risk assessment system considering these 3 genes simultaneously also did show significant correlations with VEGFA and VEGFB, some vascular stability-related genes, and hypoxia-related genes. The risk assessment system also coordinates the treatment response of the 22 drugs, which may also aid in the screening of patient treatments.

## Conclusion

In conclusion, this study used the analysis strategy of scRNA-seq and bulk RNA-seq data to reveal the cellular composition of HCC, differentiation, evolution and functional landscape of epithelial cells in HCC, provide a prognostic model of HCC, and give our insights in the detection of drug therapy for patients.

## Data Availability

The datasets presented in this study can be found in online repositories. The names of the repository/repositories and accession number(s) can be found in the article/Supplementary Material.

## Abbreviations

HCC, Hepatocellular carcinoma; scRNA-seq, Single-cell RNA sequencing; LASSO, Least Absolute Shrinkage and Selection Operator; GEO, Gene Expression Omnibus; PVTT, Portal vein tumor thrombus; TCGA, The Cancer Genome Atlas; HCCDB, Hepatocellular Carcinoma Database; UMAP, Uniform Manifold Approximation and Projection; RGE, Reversed graph embedding; DEG, Differentially expressed gene; BEAM, Branched Expression Analysis Modeling; FDR, False discovery rate; ROC, Receiver Operating Characteristic; AUC, The area under the ROC curve; EMT, Epithelial-mesenchymal transition; CDC20, Cell division cycle 20 homologue; G6PD, Glucose-6-phosphate dehydrogenase; STAT3, Signal transducer and activator of transcription 3; PLOD2, Procollagen-lysine,2-oxoglutarate 5-dioxygenase 2.
